# Comparing Laser Peripheral Iridotomy to Cataract Extraction in Narrow Angle Eyes Using Anterior Segment Optical Coherence Tomography

**DOI:** 10.1371/journal.pone.0162283

**Published:** 2016-09-08

**Authors:** Ephrem Melese, Jeffrey R. Peterson, Robert M. Feldman, Laura A. Baker, Nicholas P. Bell, Alice Z. Chuang, Lauren S. Blieden

**Affiliations:** 1 Robert Cizik Eye Clinic, Houston, Texas, United States of America; 2 Ruiz Department of Ophthalmology and Visual Science, McGovern Medical School at The University of Texas Health Science Center at Houston (UTHealth), Houston, Texas, United States of America; Justus Liebig Universitat Giessen, GERMANY

## Abstract

**Purpose:**

To evaluate the changes in anterior chamber angle (ACA) parameters in primary angle closure (PAC) spectrum eyes before and after cataract extraction (CE) and compare to the changes after laser peripheral iridotomy (LPI) using anterior segment optical coherence tomography (ASOCT).

**Methods:**

Twenty-eight PAC spectrum eyes of 18 participants who underwent CE and 34 PAC spectrum eyes of 21 participants who underwent LPI were included. ASOCT images with 3-dimensional mode angle analysis scans were taken with the CASIA SS-1000 (Tomey Corp., Nagoya, Japan) before and after CE or LPI. Mixed-effect model analysis was used to 1) compare best-corrected visual acuity, intraocular pressure, and ACA parameters before and after CE; 2) identify and estimate the effects of potential contributing factors affecting changes in ACA parameters; and 3) compare CE and LPI treatment groups.

**Results:**

The increase in average angle parameters (TISA750 and TICV750) was significantly greater after CE than LPI. TICV750 increased by 102% (2.114 [±1.203] μL) after LPI and by 174% (4.546 [± 1.582] μL) after CE (*P* < 0.001). Change of TICV750 in the CE group was significantly affected by age (*P* = 0.002), race (*P* = 0.006), and intraocular lens power (*P* = 0.037).

**Conclusions:**

CE results in greater anatomic changes in the ACA than LPI in PAC spectrum eyes. ASOCT may be used to follow anatomic changes in the angle after intervention.

## Introduction

Primary angle closure glaucoma (PACG) is a leading cause of irreversible bilateral blindness worldwide. An estimated 20 million people were affected by PACG in 2013, and this number is predicted to increase to 32 million by 2040 [1]. Angle closure is caused by either pupillary block or plateau iris, which prevents aqueous humor from leaving the eye through the trabecular meshwork, resulting in elevated intraocular pressure (IOP), either acutely or chronically, that can cause progressive, irreversible damage to the optic nerve. The primary angle closure (PAC) spectrum of disease, as defined by Foster et al [2], ranges from eyes with narrow anterior chamber angles (primary angle closure suspect [PACS]); to eyes that demonstrate clinical evidence of angle closure (PAC); to eyes with glaucomatous optic neuropathy (PACG) [2, 3]. Because the anterior chamber anatomy is a predisposing condition in the PAC spectrum, a surgical treatment to deepen the peripheral anterior chamber angle (ACA) for these eyes may prevent progression of the disease.

Laser peripheral iridotomy (LPI) is traditionally the primary mono-surgical therapy for PAC spectrum eyes [4]. Several studies have shown that the ACA in PAC spectrum eyes is significantly deepened after LPI [5–7]. *While an LPI may be sufficient to prevent progression of PAC*, several studies have shown that *>* 50% of PAC spectrum patients require further therapy for angle closure, despite anatomical angle deepening [3, 8].

In recent years, cataract surgery as a treatment for PAC spectrum diseases has been proposed [9, 10] because PAC spectrum eyes can result from thickening of the crystalline lens, which leads to the development of pupillary block and IOP elevation [11, 12]. In addition, prior studies have shown that PAC spectrum eyes have more anteriorly positioned crystalline lenses than normal eyes [13–15]. Cataract extraction (CE) with a thin intraocular lens (IOL) implantation may open the angle by deepening the ACA, repositioning the ciliary processes posteriorly, and reducing IOP, making it a potentially viable surgical treatment for PAC spectrum patients [7, 16].

Because deepening the ACA is important in the mechanism of PAC, and therefore plays a role in disease management, the ability to quantify changes in peripheral ACA anatomy after treatment for PAC spectrum disease is essential. Anterior segment optical coherence tomography (ASOCT) can be used to image the peripheral ACA consistently and reproducibly and quantify ACA parameters such as angle opening distance (AOD), trabecular-iris space area (TISA), and trabecular-iris circumference volume (TICV) [17, 18].

We previously demonstrated that both TISA and TICV increase in PAC spectrum eyes following LPI, indicating a deepening of the angle [19]. However, how these parameters change after CE and compare to LPI over the entire 360 degrees of the ACA has not been investigated. The purposes of this study are 1) to evaluate changes in ACA parameters using the swept source Fourier domain CASIA SS-1000 ASOCT (Tomey, Nagoya, Japan) before and after CE in PAC spectrum eyes; and 2) to compare the effect of CE and LPI on changes in ACA parameters in phakic PAC spectrum eyes that underwent either procedure.

## Materials and Methods

A prospective study investigating peripheral angle changes after CE in PAC spectrum eyes was conducted at the Robert Cizik Eye Clinic of the Ruiz Department of Ophthalmology and Visual Science at the McGovern Medical School at The University of Texas Health Science Center in Houston (UTHealth). Institutional Review Board approval was obtained from The University of Texas Health Science Center Committee for the Protection of Human Subjects. Written informed consent was obtained for all participants.

In addition, a retrospective study comparing the changes after CE with changes after LPI [19] (data gained from 2 prospective studies) was also conducted. The University of Texas Health Science Center Committee for the Protection of Human Subjects determined that this study was exempt from review and approved it. Both studies were HIPPA compliant and followed the tenets of the Declaration of Helsinki.

The classifications of PAC spectrum disease were adapted from Foster et al [2, 3] and defined as

- PACS: Spaeth grade A or B [20, 21] angle (defined by gonioscopy as the deepest structure visible without compression being the anterior trabecular meshwork) without any IOP-lowering medications, peripheral anterior synechiae (PAS), and glaucoma (visual field or optic nerve) damage;
- PAC: Spaeth grade A or B with IOP ≥ 21 mmHg, or on 1 or more IOP-lowering medications, or having PAS, but no glaucomatous (visual field or optic nerve) damage;
- PACG: PAC eyes with glaucomatous (visual field or optic nerve) damage.

The results of the LPI cohort have been previously published [19]. The results of the CE cohort and the comparison of the groups are presented in this paper.

### Prospective Cataract Extraction Study

#### Participants

Twenty-eight eyes of 18 participants, 45 years or older, and who were already scheduled to undergo cataract surgery with concomitant PAC spectrum disease were recruited from the Robert Cizik Eye Clinic between 2011 and 2013. Patients with anterior segment abnormalities affecting angle measurements or nanophthalmos (axial length less than 18 mm) were excluded.

#### Preoperative/Baseline Evaluation

After informed consent had been obtained, demographics (age, sex, and self-reported race) and ocular history were recorded. Participants then underwent baseline ocular evaluation. Refraction to determine the best-corrected visual acuity (BCVA) was performed using the Snellen technique. IOP was measured by Goldmann applanation tonometry, and slit lamp examination was performed. Eyes were not dilated before surgery to avoid an acute angle closure attack; therefore, the presence of a cataract was determined based on the participant’s visual symptoms and the undilated slit lamp examination.

Gonioscopy was performed using a Posner 4-mirror lens at high magnification (10x). Examination was performed without compression in dim light by experienced examiners. The angle was graded using the Spaeth gonioscopic grading scale: A = open to Schwalbe’s line; B = open anterior to trabecular meshwork; C = open to posterior trabecular meshwork; D = open to scleral spur; E = open to ciliary body band [20, 21] in each of 4 quadrants. Examination with compression was then performed to identify the presence of PAS and estimate the approximate burden of PAS as total degrees affected.

Axial length (AL), average keratometry readings (AKRs), anterior chamber depth (ACD), horizontal corneal diameter (WtW), and IOL power were obtained using the IOLMaster (Carl Zeiss Meditec Inc., Dublin, CA). ASOCT imaging was performed using the CASIA SS-1000 ASOCT in the dark (0 lux). Instrumental details and procedures for image acquisition have been previously described [18, 22]. The ASOCT operator viewed each scan to ensure that the quality was acceptable.

#### Cataract Extraction

CE was performed by temporal clear corneal phacoemulsification using a traditional divide-and-conquer technique. There were no intraoperative complications in our study population. Postoperative management and use of steroids, antibiotics, and IOP-lowering medications were at the surgeon’s discretion.

#### Follow-up

Participants were evaluated 1 month (±7 days) postoperatively. BCVA, IOP, gonioscopy, and ASOCT images were obtained a second time as described above.

#### ASOCT Image Analysis and ACA Parameters

**Fig 1:**
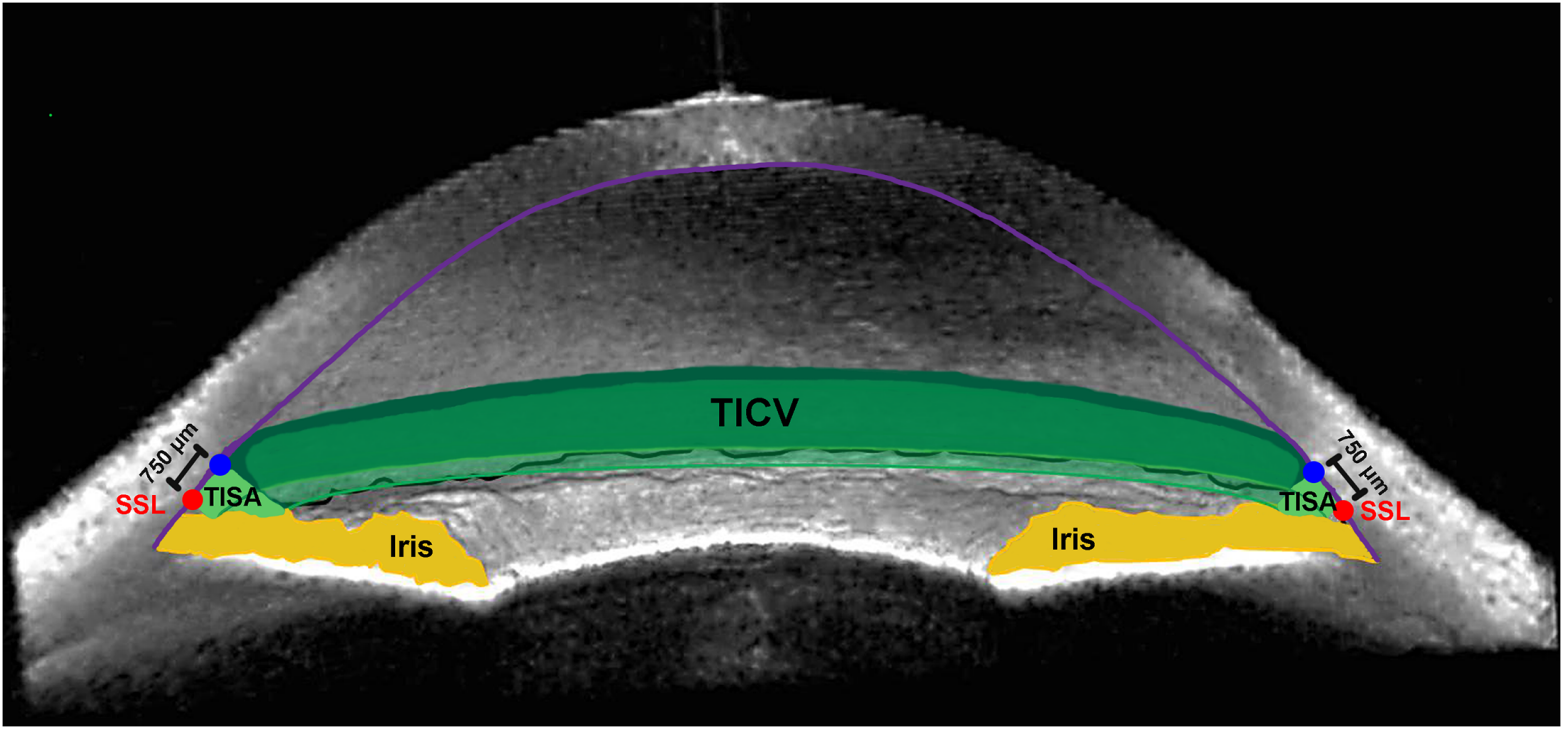
Trabecular-Iris Circumference Volume (TICV). Anterior Segment Optical Coherence Tomography (ASOCT) image exhibiting trabecular-iris space area 750 μm from the scleral spur landmark (TISA750; light green space) and trabecular-iris circumference volume 750 μm from the scleral spur landmark (TICV750; darker green spaces), along with the scleral spur landmark (red circle), iris (yellow), and cornea (violet line) in an open angle [18].

The raw ASOCT image files obtained at pre- and post-CE visits were imported into the Anterior Chamber Analysis and Interpretation software (ACAI, Houston, Texas). ACAI reading and image analysis software and methods have been previously described [18, 22]. Briefly, the scleral spur landmarks (SSLs) were identified in 16 equally spaced meridians (32 angles) by an experienced reader (LAB), who was masked to the gonioscopic grading [17]. TISA at 500 μm and 750 μm from the SSL (TISA500 and TISA750) at nasal, temporal, superior, and inferior angles, as well as TICV at 500 μm and 750 μm from the SSL (TICV500 and TICV750) were automatically calculated by ACAI (Fig 1) for images taken at baseline and 1 month after CE.

The degrees of irido-trabecular contact (ITC) were determined at baseline and 1 month after CE. The presence of ITC in each angle image was defined as the iris touching beyond 80 μm anterior to the SSL [22], which the ACAI software measured automatically in each angle. To determine the circumferential burden of ITC, 128 meridian scans (1.41 degrees between 2 consecutive angles, total of 256 angles) of each eye were obtained, and the total degrees of ITC was calculated by multiplying 1.41 degrees by the number of angles with ITC as determined by ACAI.

#### Data Analysis

Data were summarized using frequency (%) for discrete variables (i.e. race, sex, and eye). Mean and standard deviation were used for continuous variables (i.e. age, IOP, and number of IOP-lowering medications). Snellen BCVA were converted to logMAR scale, and gonioscopy grades and total PAS were converted to numerical values, i.e. Spaeth grades: A = 0, B = 1, C = 2, D = 3, E = 4; and total PAS degrees: 0–90 degrees = 1, 90–180 degrees = 2, 180–270 degrees = 3, and 270–360 degrees = 4. ACA parameters were 0 for several study eyes, as the angle was closed beyond 500 or 750 μm before CE. Thus, the percentage changes in ACA parameters before and after CE were not able to be computed for each study eye individually. The percentage change of ACA parameter reported was calculated using *mean* change divided by mean ACA parameter before CE. The Pearson correlations between TICV changes and ocular biometry parameters (AL, AKRs, ACD, and WtW) were computed.

BCVA, IOP, and ACA parameters before and after CE were compared using a mixed-effect model analysis, which takes into consideration the potential correlation between the 2 eyes in a participant. Mixed-effect model analysis was also used to identify and estimate the effects of potential contributing factors affecting changes in ACA parameters before and after CE. The random effect was the study participants; the fixed effects were age, sex, race, study eye, AL, AKRs, ACD, WtW, IOL power, and number of IOP-lowering medications at the baseline visit.

### Comparison with LPI study [19]

The results of the prospective CE study were compared with previously published data from a prospective cohort study on ACA parameter changes before and after LPI from our group [19]. Only the 34 eyes of the 21 participants who completed the pre- and post- LPI study and had acceptable ASOCT images were included for comparison. A mixed-effect model was used to compare the treatment groups (CE vs. LPI). The fixed effect was the treatment group (CE vs. LPI), and the random effect was the study participants.

All statistical analysis was performed using SAS for Window v9.4 (SAS Inc., Cary, NC), with a *P* value less than 0.05 considered statistically significant. Data are available at https://figshare.com/articles/Data_for_PONE-D-16-07110_LE_vs_LPI_xlsx/3753303.

## Results

### Prospective Cataract Extraction Study

Twenty-eight eyes of 18 participants were recruited. Fifteen (83%) females and 3 (17%) males were enrolled, and the average age was 64.8 (±6.4) years. Eight participants were (44%) White, 6 (33%) Black, and 4 (22%) Hispanic. Fourteen (50%) right eyes were included. Fifteen (54%) eyes were diagnosed as PACS, and 11 (39%) eyes were diagnosed as PAC. Five eyes (18%) had a previous patent LPI, and all eyes had a cataract on slit lamp examination. The average BCVA was 0.17 (± 0.22) logMAR, AL 22.6 (± 0.8) mm, AKRs 44.2 (± 1.6) D, ACD 2.6 (± 0.3) mm, WtW 12.1 (± 0.4) mm, and calculated IOL power 24.0 (± 3.4) D. Table 1 presents a summary of demographics and ocular characteristics, including IOP before and after the procedure and number of participants on IOP-lowering medications, for this group of patients.

**Table 1:** Summary of demographics and ocular characteristics.

Variable	CE (18 Participants, 28 eyes)	LPI (21 Participants, 34 eyes)	*P* Value
**Demographics**			
Sex, n female participants (%)	15 (83%)	16 (76%)	0.70^1^
Age, mean years ±SD	64.8 ±6.4	58.2 ±9.9	0.020^2^
Race, n participants (%)			0.62^1^
White	8 (44%)	10 (48%)	
Black	6 (33%)	4 (19%)	
Hispanic	4 (22%)	5 (24%)	
Asian	0 (0%)	2 (10%)	
**Ocular Characteristics**			
Eye, n right eyes (%)	14 (50%)	17 (50%)	1.00^1^
Primary angle closure spectrum, n eyes (%)			0.76
PACS	15 (54%)	20 (59%)	
PAC	11 (39%)	13 (38%)	
PACG	2 (7%)	1 (3%)	
Ocular Biometry Parameters			
Axial length, mean mm ±SD	22.6 ± 0.8	N/A	N/A
Average keratometry readings, mean Diopter ±SD	44.2 ± 1.6		
Anterior chamber depth, mean mm ±SD	2.6 ± 0.3		
White-to-white corneal diameter, mean mm ±SD	12.1 ± 0.4		
Calculated IOL power, mean Diopter ±SD	24.0 ± 3.4		
IOP^3^			
Pre-procedure IOP, mean mm Hg ±SD	15.6 ±2.9	16.3 ±4.1	0.49
Pre-procedure no. of IOP-lowering medications, mean ±SD and n eyes (%)	0.21 ±0.57	0.24 ±0.74	0.57
0 medication	24 (86%)	29 (85%)	
1 medication	2 (7%)	4 (12%)	
2 medications	2 (7%)	0 (0%)	
3 medications	0 (0%)	0 (0%)	
4 medications	0 (0%)	1 (3%)	
Pre-procedure no. of un-medicated eyes with IOP ≤ 21 mmHg, n eyes (%)	24 (86%)	26 (76%)	0.097
Post-procedure IOP, mean mm Hg ±SD	15.0 ±4.7	16.2 ±3.5	0.25
Post-procedure no. of IOP-lowering medications, mean ±SD and n eyes (%)	0.04 ±0.19	0.15 ±0.70	0.29
0 medication	27 (96%)	32 (94%)	
1 medication	1 (4%)	1 (3%)	
2 medications	0 (0%)	0 (0%)	
3 medications	0 (0%)	0 (0%)	
4 medications	0 (0%)	1 (3%)	
*Change in IOP*, *mean mm Hg ±SD*	*-0*.*7 ±3*.*0*	*-0*.*1 ±3*.*0*	*0*.*46*
*Change in no*. *of IOP-lowering medications*, *mean ±SD*	*-0*.*18 ±0*.*55*	*-0*.*09 ±0*.*29*	*0*.*50*
Gonioscopic Exam^3^			
Pre-procedure Spaeth gonioscopic grade^4^ at *superior* quadrant, mean ±SD and n eyes (%)	0.57 ±0.50	0.50 ±0.51	0.42
Spaeth Grade A	12 (43%)	17 (50%)	
Spaeth Grade B	16 (57%)	17 (50%)	
Spaeth Grade C	0 (0%)	0 (0%)	
Spaeth Grade D	0 (0%)	0 (0%)	
Spaeth Grade E	0 (0%)	0 (0%)	
Post-procedure Spaeth gonioscopic grade^4^ at *superior* quadrant^5^, mean ±SD and n eyes (%)	2.81 ±0.69	2.0 ±0.78	0.001
Spaeth Grade A	0 (0%)	2 (6%)	
Spaeth Grade B	1 (4%)	4 (12%)	
Spaeth Grade C	6 (23%)	20 (59%)	
Spaeth Grade D	16 (62%)	8 (24%)	
Spaeth Grade E	3 (12%)	0 (0%)	
***Change in Spaeth gonioscopic grade*, *mean ±D***	***2*.*19 ±0*.*63***	***1*.*50 ±0*.*75***	***0*.*003***
Pre-procedure PAS, degrees, mean ±SD and n eyes (%)	63.9 ±103.5	25.2 ±46.8	0.34
0–90 degrees	19 (68%)	24 (75%)	
90–180 degrees	2 (7%)	7 (22%)	
180–270 degrees	3 (11%)	1 (3%)	
270–360 degrees	4 (14%)	0 (0%)	
Post-procedure PAS^6^, degrees, mean ±SD and n eyes (%)	48.6 ±92.7	25.2 ±46.8	0.48
0–90 degrees	19 (73%)	28 (70%)	
90–180 degrees	3 (12%)	8 (20%)	
180–270 degrees	1 (4%)	4 (10%)	
270–360 degrees	3 (12%)	0 (0%)	
*Change in PAS*, *degrees*, *mean ±SD*	*-20*.*7 ±63*.*9*	*0*.*00 ±0*.*00*	*0*.*061*
BCVA			
Pre-procedure BCVA, mean logMAR ±SD	0.17 ±0.22	N/A	N/A
Post-procedure BCVA, mean logMAR ±SD	0.09 ±0.23	N/A	N/A
***Change in BCVA*, *mean logMAR ±SD***	***-0*.*08 ± 0*.*14***	***N/A***	***0*.*006***^6^

SD = standard deviation; BCVA = best-corrected visual acuity; IOP = intraocular pressure; PAS = peripheral anterior synechiae; CE = cataract extraction; LPI = laser peripheral iridotomy; IOL = intraocular lens; PACS = primary angle closure suspect; PAC = primary angle closure; PACG = primary angle closure glaucoma

^1^*P* values for sex, race, and eye were determined using a χ^2^ test.

^*2*^*P* values for age were determined using a two-sample *t*-test.

^*3*^*P* values for IOP and gonioscopic exam were determined using a mixed-effect model.

^4^A = open to Schwalbe’s line; B = open anterior to trabecular meshwork; C = open to posterior trabecular meshwork; D = open to scleral spur; E = open to ciliary body band

^5^ Postoperative gonioscopy data was not collected for 1 participant (2 eyes) in CE group

^*6*^*P* values for vision were determined using a paired *t*-test.

The correlations between TICV500 change and ocular biometry parameters were not significant (R^2^ = 0.10 and *P* = 0.099 for AL; R^2^<0.01 and *P* = 0.84 for AKRs; R^2^ = 0.06 and *P* = 0.21 for ACD; and R^2^<0.01 and *P* = 0.87 for WtW). Similarly, the correlations between the TICV750 change and ocular biometry parameters were not significant (R^2^ = 0.12 and *P* = 0.077 for AL; R^2^<0.01 and *P* = 0.87 for AKRs; R^2^ = 0.02 and *P* = 0.53 for ACD; and R^2^ = 0.01 and *P* = 0.072 for WtW).

#### Gonioscopy

Preoperative gonioscopy grades in the superior quadrant were A (43%) or B (57%) for all eyes (Table 1). Postoperatively, all eyes deepened by at least 1 grade in all quadrants, except 1 eye in the nasal quadrant and 1 eye in the inferior quadrant (Table 2). While the gonioscopy grade increased in 24 (92%) eyes after CE, only 6 (23%) eyes had a reduction in PAS from baseline (Table 3). Postoperative gonioscopy data was not collected for 1 participant (2 eyes).

**Table 2:** Spaeth Gonioscopic grading based on iris insertion at baseline and 1 month after cataract extraction (N = 26 eyes)^1^.

SpaethGrading,^2^n eyes(%)		One month after cataract extraction																			
		Nasal					Inferior					Temporal					Superior				
		A	B	C	D	E	A	B	C	D	E	A	B	C	D	E	A	B	C	D	E
Baseline	A	0(0)	1(4)	5(19)	4(15)	0(0)	0(0)	1(4)	4(15)	3(12)	0(0)	0(0)	1(4)	5(19)	3(12)	0(0)	0(0)	1(4)	4(15)	5(19)	0(0)
	B	0(0)	1(4)	1(4)	10(38)	3(12)	0(0)	0(0)	2(8)	8(31)	3(12)	0(0)	0(0)	2(8)	8(31)	3(12)	0(0)	0(0)	2(8)	11(42)	3(12)
	C	0(0)	0(0)	0(0)	1(4)	0(0)	0(0)	0(0)	0(0)	4(15)	0(0)	0(0)	0(0)	0(0)	4(15)	0(0)	0(0)	0(0)	0(0)	0(0)	0(0)
	D	0(0)	0(0)	0(0)	0(0)	0(0)	0(0)	0(0)	0(0)	1(4)	0(0)	0(0)	0(0)	0(0)	0(0)	0(0)	0(0)	0(0)	0(0)	0(0)	0(0)
	E	0(0)	0(0)	0(0)	0(0)	0(0)	0(0)	0(0)	0(0)	0(0)	0(0)	0(0)	0(0)	0(0)	0(0)	0(0)	0(0)	0(0)	0(0)	0(0)	0(0)

^1^ Missing 2 eyes from 1 participant who had gonioscopy grade A superiorly in both eyes at baseline.

^2^A = open to Schwalbe’s line; B = open anterior to trabecular meshwork; C = open to posterior trabecular meshwork; D = open to scleral spur; E = open to ciliary body band

**Table 3:** Degrees of posterior trabecular meshwork not visible and peripheral anterior synechiae at baseline and 1 month after cataract extraction (N = 26 eyes)^1^ by gonioscopy.

Degrees, n eyes(%)		One month after cataract extraction							
		PTM not visible				PAS			
		0–90	90–180	180–270	270–360	0–90	90–180	180–270	270–360
Baseline	0–90	1(4)	0(0)	0(0)	0(0)	17^2^(65)	0(0)	0(0)	0(0)
	90–180	6(23)	0(0)	0(0)	0(0)	1(4)	0(0)	1(4)	1(4)
	180–270	4(15)	0(0)	0(0)	1(4)	1(4)	1(4)	1(4)	0(0)
	270–360	10(38)	1(4)	3(12)	0(0)	1(4)	0(0)	2(8)	0(0)

^1^ 2 eyes missing follow-up data had no PAS pre-CE.

^2^1 eyes had pigment smudging at pre-CE and at 0–90 post-CE, and 2 eyes missing follow-up data had no PAS pre-CE.

PTM = posterior trabecular meshwork; CE = cataract extraction; PAS = peripheral anterior synechiae

#### ASOCT

Preoperatively, the superior angle was narrowest compared to the other angles (all *P*<0.001). There were statistically significant (*P*<0.001, Table 4) increases in TISA500 and TISA750 in all angles, with the greatest percentage increase found in the superior angle, 226% (TISA500 from 0.027 [± 0.032] mm^2^ preoperatively to 0.088 [± 0.054] mm^2^ postoperatively; Table 4). Postoperatively, there was an approximately 174% increase in both TICV500 and TICV750 values (Table 4). The total degrees of ITC had a statistically significant reduction from 211 to 72 degrees (*P*< 0.001) after CE (Table 4).

**Table 4:** Summary of anterior chamber angle parameters at baseline and 1 month after cataract extraction by anterior segment optical coherence tomography.

Variable	Baseline (N = 28 eyes)	One Month Postop (N = 28 eyes)	Change (N = 28 eyes)	% change	*P* Value
TISA500 (mm^2^, ± SD)					
Nasal	0.049 ± 0.038	0.108 ± 0.054	0.059 ± 0.039	**120**	< 0.001
Inferior	0.042 ± 0.035	0.112 ± 0.068	0.071 ± 0.044	**169**	< 0.001
Temporal	0.044 ± 0.030	0.109 ± 0.048	0.065 ± 0.040	**148**	< 0.001
Superior	0.027 ± 0.032	0.088 ± 0.054	0.061 ± 0.039	**226**	< 0.001
TISA750 (mm^2^, ± SD)					
Nasal	0.091 ± 0.063	0.218 ± 0.091	0.127 ± 0.068	**140**	< 0.001
Inferior	0.081 ± 0.064	0.227 ± 0.114	0.146 ± 0.075	**180**	< 0.001
Temporal	0.091 ± 0.050	0.217 ± 0.080	0.126 ± 0.067	**138**	< 0.001
Superior	0.061 ± 0.059	0.191 ± 0.087	0.130 ± 0.057	**213**	< 0.001
TICV500 (μl, ± SD)	1.318 ± 0.765	3.598 ± 1.377	2.280 ± 0.932	**173**	< 0.001
TICV750 (μl, ± SD)	2.619 ± 1.507	7.165 ± 2.326	4.546 ± 1.582	**174**	< 0.001
ITC (degrees, ± SD)	211 ± 95	72 ± 81	-139 ± 73	**66**	< 0.001
ITC (%, ± SD)	59 ± 26	20 ± 22	-39 ± 20	**66**	< 0.001

TISA = trabecular-iris space area; TICV = trabecular-iris circumference volume; ITC = irido-trabecular contact; SD = standard deviation

#### Factors Affecting ACA Parameters

Factors that affected change in TICV500 were age (*P* = 0.003), race (Black vs. White, *P* = 0.003), and IOL power (*P* = 0.026). TICV500 increased by 1.014 (± 0.245) μL for every decade of age and also increased more in the eyes of White patients than Black patients by 1.300 (± 0.325) μL. TICV500 decreased by 0.108 (± 0.041) μL as IOL power increased by 1 D. These same factors were found to affect changes in TICV750: age (1.837 [± 0.437] μL per decade, *P* = 0.002), race (2.052 [± 0.567] μL more in White than Black, *P* = 0.006), and IOL power (-0.174 [± 0.071] μL per Diopter, *P* = 0.037). It should be noted that baseline TICV, AL, AKRs, ACD, and WtW were not significant factors (*P*>0.21) affecting the postoperative change of TICV.

The factors affecting the change in degrees of ITC were race (*P* = 0.045), gender (*P* = 0.042), and total degrees of ITC at baseline (*P* = 0.002). The decrease in ITC was higher in males (67 [± 28] degrees) and Hispanics (vs. Black 86 [± 29] degrees). The total degrees of ITC after CE decreased 0.51 (± 0.12) degrees for every degree present at baseline.

#### IOP

The average medically treated IOP at 1-month follow-up (15.0 [± 4.7] mm Hg) was not significantly different from baseline (15.6 [± 2.9] mm Hg; *P* = 0.24). Four (14%) eyes required IOP-lowering medications preoperatively. Of these 4 eyes, 1 eye required IOP-lowering medication at 1 month after CE. No additional eyes required IOP-lowering medications (Table 1).

#### Vision

As expected, the average logMAR BCVA score significantly improved from 0.17 (± 0.22, with geometric mean approximately 20/30) at baseline to 0.09 (± 0.23, with geometric mean approximately 20/25) at 1-month follow-up (*P* = 0.006) (Table 1).

### Comparison with LPI study [19]

Comparing the present study, pre- and post- CE, with the previous study of 34 eyes of 21 participants with PAC spectrum who underwent LPI [19], there were no significant differences in sex and race between the 2 groups. However, LPI participants were significantly younger than CE participants (58.2 [±9.9] years for LPI and 64.8 [±6.4] years for CE, *P* = 0.020). Demographics and ocular examinations are summarized in Table 1 for each study and compared.

#### Comparing Gonioscopy

All eyes were graded A or B preoperatively. On average, the Spaeth grade improved by 2.19 grades in the CE group and 1.5 grades in the LPI group (*P* = 0.003). However, there were no significant differences in pre- and post-procedure PAS or change in PAS burden between groups (*P* = 0.34 for pre-procedure, *P* = 0.48 for post-procedure, *P* = 0.06 for change in PAS). Spaeth gonioscopic grading and total degrees of PAS before and after CE or LPI are summarized in Table 1.

#### Comparing ACA Parameters

**Fig 2:**
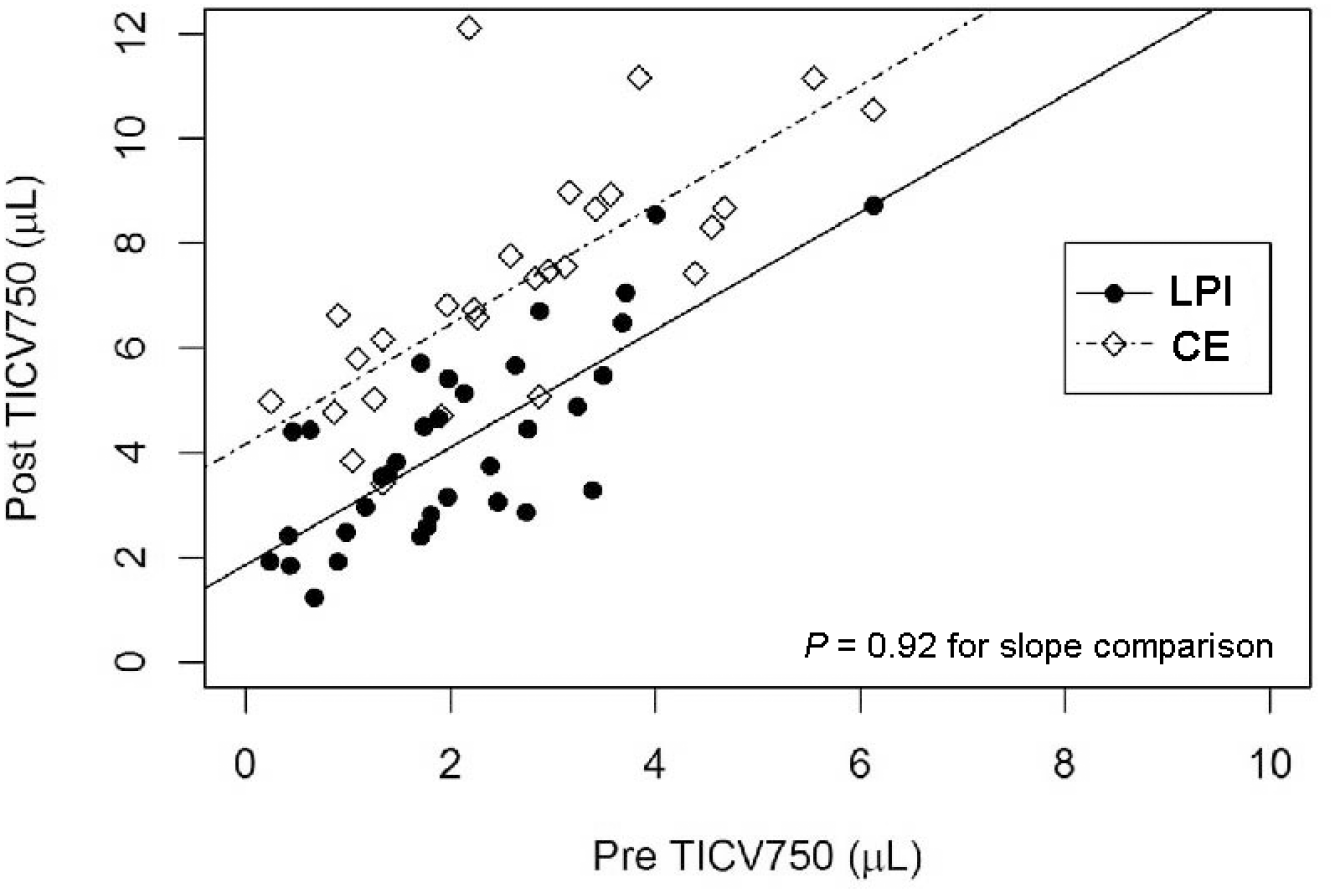
TICV750 before and after LPI and CE. Scatter plot of trabecular-iris circumference volume 750 μm from the scleral spur landmark (TICV750) before and after laser peripheral iridotomy (LPI) and cataract extraction (CE).

There were no statistically significant differences at baseline between the 2 groups, except the total degrees of ITC (*P* = 0.022). Postoperatively, all ACA parameters, except TISA750 in the nasal angle, showed a statistically significant greater magnitude of change in the CE group compared to those in the LPI group. TISA750 at the superior angle and TICV750 had the most significant differences between CE and LPI (*P*<0.001). TICV750 increased by 174% (4.546 [± 1.582] μL) after CE and by 102% (2.114 [±1.203] μL) after LPI; the magnitude of change of TICV750 was constant regardless of the baseline value (Fig 2, *P* = 0.92 for slope comparison). ACA parameters are summarized in Table 5.

**Table 5:** Comparing angle parameters and their changes before and after cataract extraction and laser peripheral iridotomy.

Variable	Baseline			Change from baseline to final visit		
	CE (28 eyes)	LPI (34 eyes)	*P* Value	CE (1 month) (28 eyes)	LPI (3 months) (34 eyes)	*P* Value
TISA 750, mean mm^2^ ± SD						
Nasal	0.091 ± 0.063	0.070 ± 0.048	0.11	0.127 ± 0.068	0.087 ± 0.072	0.058
Inferior	0.081 ± 0.064	0.053 ± 0.047	0.073	0.146 ± 0.075	0.079 ± 0.058	0.001
Temporal	0.091 ± 0.050	0.081 ± 0.047	0.32	0.126 ± 0.067	0.067 ± 0.055	0.004
Superior	0.061 ± 0.059	0.036 ± 0.034	0.068	0.130 ± 0.057	0.047 ± 0.044	<0.001
TICV750, mean μL ± SD	2.619 ± 1.507	2.065 ± 1.274	0.11	4.546 ± 1.582	2.114 ± 1.203	<0.001
Total degrees of ITC, mean degrees ± SD	211 ± 95	268 ± 69	0.022	-139 ± 73	-56 ± 95	0.002

CE = cataract extraction; LPI = laser peripheral iridotomy; TISA750 = trabecular-iris space area 750 μm from the scleral spur landmark; TICV750 = trabecular-iris circumference volume 750 μm from the scleral spur landmark; SD = standard deviation

#### Comparing IOP

Preoperatively, the majority of participants in these 2 cohorts did not have elevated IOP (> 21 mmHg). Twenty-four CE eyes (86%) and 26 LPI eyes (76%) had IOP ≤ 21 mmHg without using any IOP-lowering medications. The average IOP reduction from baseline to the final follow-up visit was similar between CE and LPI (0.7 [± 3.0] mm Hg for CE and 0.1 [± 3.0] mm Hg for LPI; *P* = 0.46). A total of 9 eyes (4 [14%] eyes for CE and 5 [15%] eyes for LPI) required IOP-lowering medications preoperatively. Of the 4 CE eyes, 1 eye still required IOP-lowering medication at 1 month after CE and of the 5 LPI eyes, 2 eyes still required IOP-lowering medications 3 months after LPI (Table 1).

## Discussion

Our study demonstrated significant increases in ACA parameters, including TISA500, TISA750, TICV500, TICV750, and extent of ITC, after CE in participants with concomitant PAC spectrum disease. These results are consistent with the angle deepening demonstrated by the change in gonioscopic grading for the majority of participants. We also found that all ACA parameters increased significantly more in PAC spectrum eyes that underwent CE compared to those receiving LPI, except for TISA750 at the nasal angle, which showed the same trend but did not achieve statistical significance (*P* = 0.058).

Many studies have used ASOCT to image the angle before and after LPI [6, 23] as well as CE [7, 24, 25], but all of these studies quantify ACA parameter changes only in horizontal angles (nasal and temporal) using linear or 2-dimensional measurements (AOD or TISA). This is the first study to quantify angle opening over the entire 360 degrees of the angle, using a 3-dimensional volume measurement (TICV) and circumferential measurement (extent of ITC) after CE. It is also the first study to demonstrate that CE results in a significantly greater effect in 3-dimensional ACA parameters compared to LPI (confirmed via a PubMed search on August 8, 2016, using search terms phacoemulsification, angle closure, peripheral iridotomy, and cataract).

### Changes in ACA Parameters

CE resulted in statistically significant increases in TISA in all angles. The superior angle was the narrowest before CE and also showed the largest percentage increase in TISA after CE. This is not surprising as the superior angle is typically the narrowest [18, 22, 26]. Other studies have assessed the effect of CE on TISA in PAC spectrum patients and have also shown increases in TISA postoperatively [7, 27–29]. Finally, while our previous study showed that LPI does increase TISA values in PAC spectrum eyes [19], our comparison showed that CE produced a significantly greater change in TISA values when compared to the effect seen with LPI.

Similarly, CE also showed a statistically greater change in TICV values over that of LPI. We recently described TICV as a novel and reliable 3-dimensional ACA parameter [18]. TICV integrates the volume of the entire peripheral angle at a defined distance from the SSL ring, and we have recently presented the TICV750 threshold values for determining a narrow or open angle (4.23 μL) [30]. We found that all but 2 eyes of 1 participant crossed this threshold with postoperative TICV750 values greater than 4.23 μL after CE. The 2 eyes that did not meet the TICV750 threshold had a significant amount of PAS (> 180 degrees). This finding may reflect a normalization of TICV value in pseudophakic PAC spectrum eyes, with a resultant opening of the angle, and highlights the significant contribution of the crystalline lens to PAC.

### PAS

There was no difference in PAS burden after CE, and this was consistent when compared to the LPI data, supporting the idea that CE and LPI do not treat PAS. It has been reported previously that the amount of PAS remains stable post-LPI [19, 31], but the impact of CE on PAS remains controversial [10, 32]. It is important to note we made no specific attempts (i.e. goniosynechiolysis) to physically open the angle during CE. However, surgical technique during CE (e.g. amount and type of viscoelastic used, use of iris expansion devices, differing fluid dynamics) may release PAS with varying effect. This is beyond the scope of this study and needs further investigation.

Because it is not possible to differentiate PAS from appositional closure without adhesion on ASOCT, ITC was graded on imaging pre- and post- CE. Although we did not find a significant decrease in the burden of PAS gonioscopically after either CE or LPI, we found a reduction in the total degrees of ITC detected by ASOCT after both CE (*P*<0.001) and LPI (*P* = 0.011). The reduction of total degrees of ITC after CE was significantly more than the reduction after LPI (*P* = 0.002).

### IOP

In contrast to the published literature [33, 34], our study did not find a significant change in IOP after CE. In fact, when comparing the effect of CE to LPI, neither group showed a significant change in IOP. To preface the discussion, IOP was not a primary outcome in our study; therefore, our study was not powered to detect changes in IOP. However, there are 2 points to be considered: 1) the immediate lowering of IOP from baseline; and 2) the overall long-term control of IOP in PAC spectrum eyes. To the first point, the discrepancy between our study and prior publications on the effect on IOP after either CE or LPI is probably due to differences in baseline IOP, which was always a treated IOP in our population. Shams et al showed that baseline IOP was an independent predictor of postoperative IOP after CE in patients with angle closure, meaning greater changes in IOP were seen with higher baseline IOP [34]. Since fewer patients in our study had poorly controlled IOP at baseline because they were being treated, we may have seen less of an effect on IOP postoperatively. We did, however, see a reduction in IOP-lowering medications in the CE study. Additionally, the inclusion of PAC suspects, who by definition have normal IOP and are not on IOP-lowering medications, may have diluted the result. Notably, at least in our study population, pre-treatment IOP and pre-treatment TICV were not correlated nor was the change in TICV with the IOP. The change in ITC from either intervention also did not correlate with IOP. Regarding long-term IOP control, our follow-up periods were too short to comment on how either intervention ultimately affects long-term IOP control in PAC eyes.

### Factors affecting Changes in TICV

#### Age

In the CE portion of our study, we found that the magnitude of change in TICV after cataract extraction increased with age. This observation reflects the known age-related steepening of anterior curvature and increase in thickness of the lens [35]. We did not see this effect of age on TICV750 in our LPI group, which was younger with a narrow and limited range, so age may act as a confounder when analyzing the changes in TICV750 between groups (mean difference = 2.432 μL without adjusting for age), resulting in an overstated effect of CE. However, after adjusting for the age effect (as covariate), the difference in the effect of intervention on TICV750 values between CE and LPI remained (adjusted mean difference was 1.936 μL; *P*<0.001). Importantly, there was a significant age difference between treatment groups, which is expected as the incidence of cataract development increases with age. The expected effect of age on TICV750 is -0.76 μL per decade, with no difference between 55–65 and 65–79 age groups in normal eyes [18]. Thus the effect of age on these results should be limited, although this is a limitation of our study and is partially due to the controversial role of clear lens extraction in patients with PAC spectrum. In addition to the effect of age on TICV described above, it should be noted that the underlying mechanisms of anatomic angle closure may be different between older patients with cataracts and younger patients with clear lenses and warrants further investigation.

#### IOL power

Interestingly, in our CE patients, we found an inverse relationship between implanted IOL power and magnitude of change in TICV. To attempt to understand this correlation, we evaluated various biometric properties of the eye (AL, corneal curvature, ACD, and horizontal WtW measurements). None of the factors contributing to the determination of IOL power were independently significant. We believe that because of our small study population, IOL power is likely a composite surrogate for all these biometric factors. A larger study may be able to determine which biometric properties would more greatly affect TICV values and angle anatomy.

#### Race

In our CE participants, ITC and TICV were found to be affected by race (Black vs. White). A majority of the published studies investigate angle closure in Asians. Because our study did not include Asian participants, we cannot interpret our results in the context of these other studies. Our results highlight the potential importance of race on changes in ACA parameters, and this topic deserves further study.

### Limitations

Aside from the limitations discussed above, another consideration is that comparison between the CE and LPI studies is a retrospective review of 2 cohort studies, and the sample size was predetermined by those 2 studies. However, a *post hoc* power calculation for change in TISA750 and TICV750 was greater than 0.99, except for TISA750 nasally (power = 0.73), for detecting a 0.05 mm^2^ difference in change in TISA750 between the 2 groups and a 1 μL difference in TICV750 using a mixed-effect model. This lack of power may be responsible for the inability to detect a statistically significant difference in TISA750 in the nasal angle. Our study is also limited by differences in follow-up time between CE (1 month) and LPI (3 months) groups. Another potential limitation is that we used both eyes from a participant if eligible instead of only one eye. As we are comparing imaging outcomes instead of clinical outcomes, we believe the effects on the results of this study are likely minimal. To minimize this potential effect, we used a mixed-effect model, which takes into consideration the correlation between eyes in a participant. However, it is possible that the correlation may not be completed eliminated. Additionally, long-term studies are needed to determine if changes in angle anatomy are maintained and if these changes correlate with the clinical control of glaucoma.

We were unable to measure lens thickness due to instrument availability. Given the age difference between the 2 groups, it is unclear whether this may be of interest as we know that the development of a cataract increases lens thickness. We did find that preoperative ACD in the CE patients did not affect change in TICV. The relationship of the central anterior chamber to the peripheral anatomy is complex, and we cannot assume that the mechanisms of PAC in younger patients with clear lenses are the same as older patients with cataracts. This deserves further study in a prospective manner.

In conclusion, CE is a powerful treatment option in patients with PAC spectrum disease, which results in not only in significant opening of the angle but improved visual acuity. We also have found that CE results in a greater opening of the ACA than LPI as measured by TICV. This study lends further anatomic support to the emerging notion of CE as an effective treatment for PAC. Qualitative gonioscopic documentation of the change in angle width and subsequent follow-up after surgical treatment may be supplemented with objective means, such as ASOCT imaging with 3-dimensional parameters.
